# Immune cells are associated with mortality: the Health and Retirement Study

**DOI:** 10.3389/fimmu.2023.1280144

**Published:** 2023-10-20

**Authors:** Gokul Seshadri, Sithara Vivek, Anna Prizment, Eileen M. Crimmins, Eric T. Klopack, Jessica Faul, Weihua Guan, Helen C. S. Meier, Bharat Thyagarajan

**Affiliations:** ^1^Department of Laboratory Medicine and Pathology, University of Minnesota, Minneapolis, MN, United States; ^2^Leonard Davis School of Gerontology, University of Southern California, Los Angeles, CA, United States; ^3^Survey Research Center, Institute for Social Research, University of Michigan, Ann Arbor, MI, United States; ^4^Division of Biostatistics, School of Public Health, University of Minnesota, Minneapolis, MN, United States

**Keywords:** T-cells, B-cells, dendritic cells, NK cells, neutrophils, inflammation, mortality, Health and Retirement Study

## Abstract

**Introduction:**

Age-related immunosenescence is characterized by changes in immune cell subsets and is associated with mortality. However, since immunosenescence is associated with other concurrent age-related changes such as inflammation and multi-organ dysfunction, it is unclear whether the association between age-related immunosenescence and mortality is independent of other concurrent age-related changes. To address these limitations, we evaluated the independent association between immune cell subsets and mortality after adjustment for age-related inflammation and biologic age.

**Methods:**

Data for this study was obtained from the 2016 interview of the Health and Retirement Study (N=6802). Cox proportional hazards regression models were used to estimate the association between 25 immune cell subsets (11 T-cell subsets, 4 B-cell subsets, 3 monocyte subsets, 3 natural killer cell subsets, 3 dendritic cell subsets, and neutrophils) and 4-year mortality adjusting for covariates such as the Klemera-Doubal algorithm biological age, chronological age, gender, race/ethnicity, BMI, smoking status, comorbidity index, CMV seropositivity, and inflammatory latent variable comprising C-reactive protein, and 4 cytokines (interleukin-10, interleukin-1 receptor antagonist, interleukin-6, and soluble tumor necrosis factor).

**Results:**

Four hundred and seventy-six participants died during the study period with an overall median follow up time of 2.5 years. After controlling for covariates and adjustment for sample-weights, total T cells [HR: 0.86, p=0.004], NK CD56LO cells [HR: 0.88, p=0.005], and neutrophils [HR: 1.22, p=0.004] were significantly associated with mortality.

**Conclusions:**

These findings support the idea that an aging immune system is associated with short-term mortality independent of age-related inflammation or other age-related measures of physiological dysfunction. If replicated in other external cohorts, these findings could identify novel targets for both monitoring and intervention to reduce the age-related mortality.

## 1 Introduction

Aging is a complex biological process, characterized by a decline in the physiological functioning of several organ systems. Chronological age remains the strongest odds factor for increased susceptibility to various diseases, and is associated with higher incidence and prevalence of chronic conditions in older individuals (1). However, chronological age is a non-modifiable odds factor and several researchers have developed multidimensional aging constructs (e.g.) Klemara Doubal biological age (KDM-BA) (2), phenotypic age (3) and epigenetic age (4) that better reflect the age-related physiologic dysfunction in older individuals and are associated with mortality even after adjustment for chronological age (5).

The aging immune system, commonly referred to as immunosenescence represents another aspect of biological aging that has not been extensively studied in the context of the multi-dimensional aging constructs. Immune aging is characterized by a reduction in naïve T cells and an accumulation of memory T cells, lower antibody production and lower natural killer cell cytotoxicity (6–8). Studies have shown that age-related changes in the immune system (8–10) contribute to an age-related immunosenescence (11, 12). We have previously reported that low CD4+ naïve T cell counts was associated with KDM-BA, multimorbidity and mortality in the Health and Retirement Study (HRS), a nationally representative study of adults over aged 50 years (13). Features of immunosenescence are consistently associated with mortality across a range of study populations. For example, another study among older adults (14) showed that the absolute number of CD8+ memory T cells correlated with increased mortality after adjustment for chronological age. A low percentage of total lymphocytes was also found to be associated with overall mortality in patients with sepsis (15). Furthermore, naïve T cell depletion and increased percentage of CD8+ central-memory T cells were shown to be associated with all-cause mortality in hemodialysis patients (16). A single center study on hemodialysis patients in Spain (17) showed that B-cells count <100 (HR: 2, 95% CI: 1.05–3.8, p=0.03) and NK cells count < 60 (HR: 2.4, 95% CI: 1.06–5.5, p=0.03) were associated with higher odds of all-cause mortality. In addition, a retrospective study of 163 COVID-19 patients greater than 18 years of age showed that all-cause mortality was associated with lower levels of lymphocytes and NK cells, and higher levels of leukocytes, neutrophils, and high sensitivity CRP (18).

Since the multidimensional aging constructs are strongly correlated with chronological age, and the co-morbidities are also correlated with each other, it is unknown if immunosenescence and these multidimensional aging constructs are tapping into related aging processes contributing to increased mortality odds. In addition, aging is associated with a chronic low-grade inflammatory state known as “inflammaging” (1, 19–21). Persistent inflammation can contribute to cell damage and is implicated in the etiology and progression of various age-related conditions, including cardiovascular disease (22, 23) and neurodegenerative disorders (24, 25). Though the exact cause of inflammaging and accelerated biological age remains unclear (26), it is thought that age-related changes in the distribution of immune cells and the abnormal functioning of the immune system may both contribute to inflammaging and accelerated biological aging. Hence, while both immunosenescence and inflammaging are thought to be contributors to the pathogenesis of age-related diseases, previous studies have not evaluated whether the association between immunosenescence and mortality is independent of inflammaging. To address this knowledge gap, we examined whether features of immunosenescence (adaptive and innate immune cell subsets) were associated with mortality independent of a multi-dimensional aging construct (KDM-BA), co-morbidities and inflammaging.

## 2 Methods

### 2.1 Study population

The Health and Retirement Study (HRS) is a national longitudinal study of older Americans, with support and funding from the National Institute on Aging and the Social Security Administration. Since its inception in 1992, the HRS has conducted biennial surveys, and replenished the cohort every 6 years (27, 28). The HRS uses a multistage area probability sample design coupled with oversampling of special populations (Blacks, Hispanic, and residents of Florida), and sample weights are used to compensate for the unequal probabilities (29).

Venous blood samples were collected from community dwelling HRS participants who were not new respondents and who completed their own interview in 2016, and immune cell subsets were measured among 9933 participants. This study includes only those participants with age greater than 55 during 2016 wave, and after excluding participants with missing values in age, sex, race/ethnicity, body mass index (BMI), smoking status, and immune cell data, and then accounting for subsample weights, data from 6802 participants (6326 participants who were alive and 476 participants who died over the 4-year follow until 2020), were included in this study. The age range for this study ranged from 56 years to 90 years.

### 2.2 Primary outcome

Mortality was assessed at the 2020 interview by those who provided a report of death of the HRS participant. This mortality variable and reported date of death were used to compute survival time for each participant in our dataset with their 2016 Venous Blood Study sample collection date as the baseline.

### 2.3 Primary exposure variables

In the Health and Retirement Study, immune cell subsets were estimated using multiparameter flow cytometry performed on cryopreserved Peripheral Blood Mononuclear Cells (PBMC) processed from blood samples (30). Twenty-four immune subsets including 11 T-cells, 4 B-cells, 3 monocytes, 3 NK cells, and 3 dendritic cells were obtained using an LSRII and a FORTESSA X20 flow cytometers. Flow cytometry results were analyzed using OpenCyto by applying hierarchical gating strategies, which are then reviewed with FlowAnnotator and further refined using FlowJo software (FlowJo LLC, Ashland, OR) if necessary (31). Neutrophils were obtained from the Complete Blood Count (CBC) performed on the whole blood using a Sysmex XE-2100 instrument.

### 2.4 Cryopreservation protocol

Cryopreservation, thawing, and immunophenotyping protocols used in the HRS have been published previously (30). More than 95% of the blood samples collected in CPT tubes were processed within 48 hours of sample collection and all samples were centrifuged and processed within 72 hours of sample collection. The PBMC cryovials were stored in a Styrofoam container at -80° C to maintain a controlled cooling rate. Cold 20% DMSO (in 1× RPMI) solution and cold 20% FBS (in 1× RPMI) were used to process the PBMCs. PBMCs were stored in liquid nitrogen freezer allowing evaluation of differences in subset recovery or viability due to changes in temperature. Fast thawing was employed, PBMCs were thawed at 37° C for 60 seconds. Cells were rested for 1 hour at 37°C after thawing to optimize the subsequent immunophenotyping, staining protocols.

### 2.5 Immunophenotyping

The markers used to determine 24 immune cell subsets are described in **Supplementary Table 1**. Immune cell subsets were represented as percentages of their parent population (32). Total T-cells and B-cells were expressed as a percentage of total lymphocytes. CD4+ and CD8+ subsets of T-cells were expressed as percentage of T-cells. Subsets of CD4+ and CD8+ cells (effector, effector memory, central memory and naïve cells) were expressed as percentage of CD4+ and CD8+ cells respectively. Subsets of B-cells were expressed as percentage of B-cells. Monocytes, DCs and NK cells were expressed as a percentage of PBMCs. Subsets of monocytes, DCs and NK cells were expressed as a percentage of monocytes, DCs and NK cells respectively. We have previously shown that immunophenotyping of cryopreserved PBMCs using protocols used in HRS show cell percentages similar to those observed in blood samples without cryopreservation (32).

### 2.6 Gating strategy

Gating strategies used in HRS have been described in detail in a previous publications (31, 32) and are shown in **Supplementary Figures 1A, B**. The 24 immune cell subsets were measured using two flow cytometry panels.

Panel 1 (lymphocyte panel): Briefly, lymphocytes were initially gated on an FSC-A/SSC-A dot plot. Among the lymphocytes identified by the FSC-A/SSC-A dot plot, single cells were selected on an FSC-W/FSC-H dot plot. Live single lymphocytes were then gated on viability dye/SSC-A dot plot. T cells (CD3+ CD19−) and B cells (CD3− CD19+) were gated on a CD3/CD19 dot plot. B cells were further gated on an IgD/CD27 dot plot into three subsets: IgD+ memory B cells (CD3− CD19+ CD27+ IgD+), IgD− memory B cells (CD3− CD19+ CD27+ IgD−) and naïve B cells (CD3− CD19+ CD27−). T cells were divided into cytotoxic T cells (CD4− CD8+) and helper T cells (CD4+ CD8−) using a CD4/CD8 dot plot. Both cytotoxic and helper T cells were gated into four subsets using a CCR7/CD45RA dot plot: Effector (EFF, CD45RA+, CCR7−), Effector memory (EM, CD45RA−, CCR7−), Central memory (CM, CD45RA−, CCR7+) and Naïve (N, CD45RA+, CCR7+) cytotoxic or helper T cell subsets.

Panel 2 (PBMC panel): Monocytes, dendritic cells (DC) and natural killer (NK) cells were identified using a second panel of antibodies. After identifying single live cells as described above, we used a CD3/CD19 dot plot and selected CD3− CD19− live single PBMCs to identify DC, NK and monocytes. Monocytes were characterized as CD14+ cells using a CD14/CD20 dot plot. A subsequent HLA-DR/SSC-A dot plot further identified the monocytes (removing potential NK cells contamination) as CD14+ HLA-DR+ monocytes. Lastly total monocytes were divided into CD16+ and CD16− monocytes on a CD16/CD14 dot plot. NK cells were identified as CD14− CD20− cells on a CD20/CD14 dot plot. NK cells were further characterized as CD16+ using a CD16/SSC-A dot plot and NK cells subsets were defined as CD56HI (CD56++) and CD56LO (CD56+) on a CD56/CD16 dot plot. DCs were identified as CD14− CD20− cells on a CD20/CD14 dot plot. Subsequently, an HLA-DR/SSC-A dot plot was used to differentiate HLA-DR+ DCs from NK cells. DCs were further dived into two subsets, plasmacytoid DC (CD123+CD11c−) and myeloid DCs (CD123− CD11c+) on a CD123/CD11c dot plot.

### 2.7 Measurement of covariates

Chronological age (in years), gender (female/male), and race/ethnicity (Hispanic White, Non-Hispanic White, Non-Hispanic Black, and Non-Hispanic Other) were obtained from the HRS demographics data (28). BMI was calculated from height and weight measured during physical examinations conducted at 2014 and 2016 (28). Smoking status was self-reported and categorized as never smokers, former smokers, and current smokers. Cytomegalovirus (CMV) seroprevalence was measured quantitatively with IgG antibodies in serum using the Roche e411 immunoassay analyzer, the results were dichotomized in this analysis as positive (reactive) and negative (non-reactive and borderline). High sensitivity C-reactive protein (CRP) was measured in serum using a latex-particle enhanced immunoturbidimetric assay kit and read on the Roche COBAS 6000 Chemistry analyzer. Comorbidity index was created by counting the number of self-reported chronic conditions in the 2016 HRS survey. The chronic conditions used to estimate the comorbidity index included hypertension, type II diabetes, cancer, lung disease, cardiac disorders, stroke, arthritis, and psychiatric problems. The presence of hypertension, and type II diabetes were determined by blood pressure measurements and HbA1c measurements respectively and by the use of medications at 2016 interview. Cancer, lung disease, cardiac disorders, stroke, arthritis, and psychiatric problems were self-reported. Interleukin-1 receptor antagonist (IL-1RA), Interleukin-6 (IL-6), Interleukin-10 (IL-10) and soluble Tumor Necrosis Factor (sTNFR-1) were measured in serum using the respective Simple Plex assays on the ELLA System (33). A latent variable was created from log transformed values of CRP, IL-1RA, IL-10, sTNFR-1 and IL-6 using confirmatory factor analysis in MPlus v8 to represent systemic inflammation (34).

### 2.8 KDM biological age

Klemara Doubal’s method (KDM) (2) was used to compute biological age which captures age-related physiological decline and mortality odds using a set of biomarkers: systolic blood pressure (cardiac function), total cholesterol and fasting glucose (metabolic markers), CMV and CRP (inflammation), serum creatinine and blood urea nitrogen (kidney function), alkaline phosphatase and albumin (liver function), peak expiratory flow (lung function). Biological age was calculated using the function ‘kdm_calc’ of the R package ‘BioAge’.

## 3 Statistical analysis

**Figure 1:**
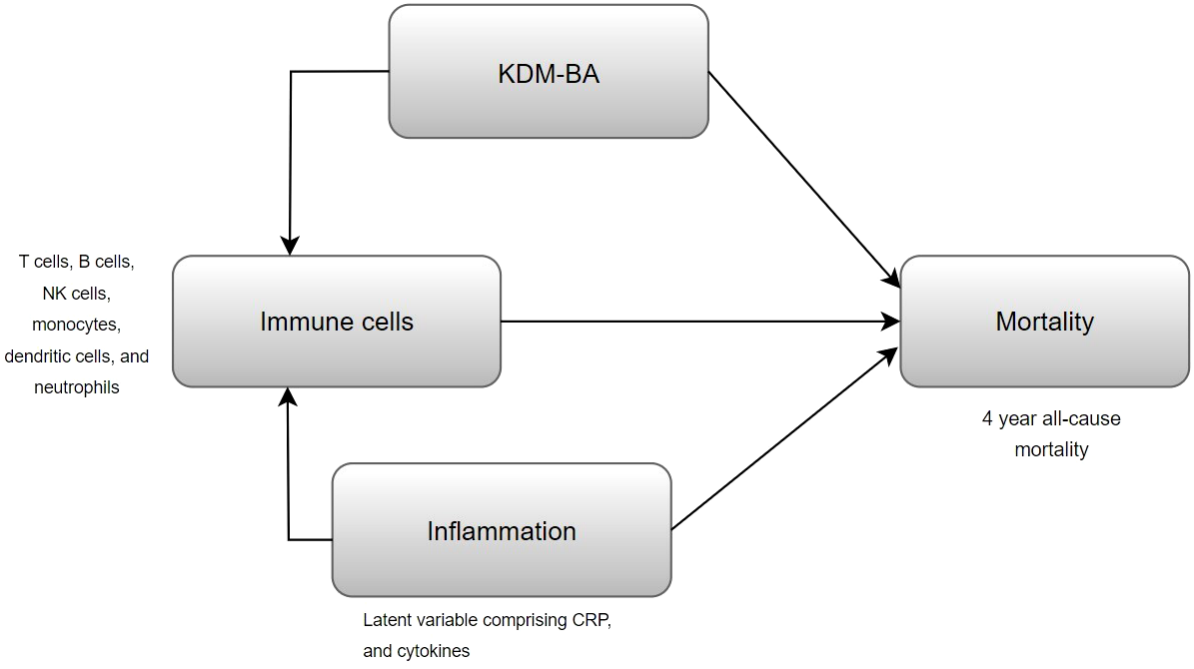
Conceptual diagram of the performed analysis, evaluating whether KDM-BA or inflammation confounds the association of immune cells with mortality.

The primary objective of this study was to identify immune cell subsets that were associated with mortality after adjustment for KDM-BA (**Figure 1**). Our secondary aim was to evaluate whether inflammation confounded the association between immune cell subsets and mortality (**Figure 1**). We evaluated differences in population characteristics between those who were alive and those who died between 2016 and 2020 using two sample t tests for continuous covariates and chi-squared test for categorical covariates. Participant weights were defined in HRS to make the data representative of the US population over the age of 55 and to account for over sampling of Blacks and Hispanics. All predictor variables and covariates were standardized to have a mean of 0 and a standard deviation of 1, such that hazard ratios would correspond to one standard deviation increase in immune cells. Survival time (in days), calculated from blood sample collection and reported date of death (censored on 12/31/2020 for those who were alive), was used as the time to event in our survival analysis. To study associations between individual immune cells and mortality, survey-weighted Cox proportional hazards regression models were used after adjustment for chronological age, gender, race/ethnicity, BMI, smoking status, and CMV seropositivity (Model 1). We additionally adjusted for KDM-BA and comorbidity index, to see if associations were independent of multi-dimensional aging construct (KDM-BA) and multi-morbidities (Model 2). Subsequently, we controlled for systemic inflammation (Model 3) using a latent variable derived from CRP and cytokines (IL-6, IL-10, IL-1RA, sTNF-1) to evaluate whether inflammation confounded the association between immune cells and mortality. All the Cox proportional hazards models were weighted by subsample weights to account for complex survey design used in HRS. Benjamini-Hochberg’s correction procedure was applied on models’ results to control the inflated false discovery rates due to multiple testing.

All analyses were performed using Python 3.9.13 and R version 4.2.2.

## 4 Results

### 4.1 Overview of participant characteristics

Among the 6802 participants included in this study, 476 participants (6.99%) died over 4 years of follow up. Distribution of study variables measured in 2016 for those who were either dead or alive in 2020 (over 4 years of follow up) in our study cohort is shown in **Table 1**. As expected, participants who died during the follow up period were significantly older than those who were alive at the end of the follow up period in 2020 (77.1 years vs. 66.9 years; p<0.01). Participants who were alive were more likely to be Non-Hispanic White, women, never smokers, have lower CRP, lower comorbidity index and also more likely to be overweight/obese as compared to participants who died during the follow up period (**Table 1**).

**Table 1:** Descriptive statistics for alive and dead subgroups of 6802 Health and Retirement Study participants measured in 2016.

Variables	Alive (6326)	Dead (476)	P-Values
Biological Age (KDM-BA *years*), mean (SD)	66.9 (8.8)	77.1 (9.5)	<0.01
AGE (*years*), mean (SD)	67.3 (8.0)	75.64 (9.3)	<0.01
Total Survival Time (*days*), mean (SD)	1541.7 (103.9)	921.1 (456.8)	<0.01
Gender, n (%) Women	3363 (53.2)	249 (52.3)	0.73
RACE, n (%)			0.76
Hispanic White	313 (4.9)	24 (5.0)	
Non-Hispanic Black	584 (9.2)	50 (10.5)	
Non-Hispanic Other	207 (3.3)	15 (3.2)	
Non-Hispanic White	5222 (82.6)	387 (81.3)	
CMV Status, n (%) Positive	3842 (60.7)	342 (71.8)	<0.01
C-Reactive Protein (*mg*/*L*), mean (SD)	3.3 (4.0)	4.4 (4.9)	<0.01
Comorbidity Index, mean (SD)	2.1 (1.4)	2.9 (1.5)	<0.01
Smoking Status, n (%)			0.01
Current smokers	745 (11.8)	77 (16.2)	
Former smokers	2760 (43.6)	227 (47.7)	
Never smokers	2821 (44.6)	172 (36.1)	
Body Mass Index (*Kg*/*m*^2^), mean (SD)	28.5 (5.7)	28.1 (7.0)	0.32
lnterleukin-10 (*pg*/*mL*), mean (SD)	3.8 (6.7)	4.7 (7.5)	0.03
Interleukin-1 receptor antagonist (*pg*/*mL*), mean (SD)	565.3 (389.3)	692.9 (1127.3)	0.13
lnterleukin-6 (*pg*/*mL*), mean (SD)	6.9 (57.2)	15.8 (160.4)	0.25
(soluble) Tumor Necrosis Factor (*pg*/*mL*), mean (SD)	1643.1 (564.9)	2126.4 (1220.8)	<0.01

### 4.2 Association between immune cells and mortality

After adjusting for chronological age, gender, race/ethnicity, smoking status, CMV seropositivity, and BMI (**Table 2** Model 1), one standard deviation increases in percentage of total T cells, CD4+ Tn, and NK LO subsets were associated with a 18% (p=0.0005), 12% (p=0.04), and 13% (p=0.002) reduced odds of mortality respectively. In addition, one standard deviation increase in DC-M was associated with a 9% reduction in mortality though this association was not statistically significant (p=0.10) (**Table 2**). One standard deviation increases in CD4+, Tem, IgD- Mem B, and neutrophils were associated with 12% (p=0.004), 9% (p=0.049) and 36% (p<0.0001) increased mortality odds respectively (**Table 2**). After additionally adjusting for comorbidity index and KDM-BA (**Table 2** Model 2), most of the associations seen in Model 1 remained unchanged with two exceptions; CD4+ Tn was no longer significantly associated with mortality (9% decrease; p= 0.09) and DC-M became significantly associated with mortality (12% decrease; p = 0.02) (**Table 2**). A one standard deviation increase in percentage of total T cells, DC-M, and NK LO subsets was associated with a 16% (p=0.001), 12% (p=0.02), and 13% (p=0.002) reduced odds of mortality respectively, similarly CD4+ Tem, IgD- Mem B, and neutrophils were associated with 11% (p=0.01), 9% (p=0.03) and 29% (p=0.0003) increased mortality odds respectively (**Table 2**).

**Table 2:** Association between immune cell subsets and mortality in the Health and Retirement Study.

Immune cell subsets	Model 1: [HR (95% CI); p-value]	Model 2: [HR (95% CI); p-value]	Model 3: [HR (95% CI); p-value]
T total	0.82 (0.74-0.92); p = 0.0005*	0.84 (0.75-0.93); p = 0.001*	0.86 (0.77-0.95); p = 0.004*
CD4+ T naïve	0.88 (0.78-0.99); p = 0.04	0.91 (0.81-1.01); p = 0.09	0.92 (0.82-1.03); P = 0.14
CD4+ Tem	1.12 (1.04-1.22); p = 0.004*	1.11 (1.02-1.20); p = 0.01	1.11 (1.02-1.21); p = 0.01
IgD- mem B	1.09 (1.00-1.18); p = 0.049	1.09 (1.00-1.19); p = 0.03	1.09 (1.00-1.19); p = 0.03
NK LO	0.87 (0.79-0.95); p = 0.002*	0.87 (0.79-0.95); p = 0.002*	0.88 (0.80-0.96); p = 0.005*
DC-M	0.91 (0.82-1.01); p = 0.10	0.88 (0.79-0.98); p = 0.02	0.87 (0.79-0.97); p = 0.01
Neutrophils	1.36 (1.18-1.56); p < 0.0001*	1.29 (1.12-1.48); p = 0.0003*	1.22 (1.06-1.40); p = 0.004*

Model 1: Adjusted for chronological age, gender, race/ethnicity, smoking status, CMV seropositivity, and BMI.

Model 2: Adjusted for covariates in Model 1 + KDM + comorbidity index.

Model 3: Adjusted for covariates in Model 2 + inflammation latent variable.

*p value that is significant after Benjamini-Hochberg correction.

The associations between 6 immune cell subsets and mortality (from **Table 2** Model 2) remained unchanged with additional adjustment for the inflammation latent variable (**Table 2** Model 3). A one standard deviation increase in percentage of T total, DC-M, and NK LO subsets was associated with a 15% (p=0.004), 13% (p=0.01), and 12% (p=0.005) reduced odds of mortality respectively, similarly CD4+ Tem, IgD- Mem B, and neutrophils were associated with 11% (p=0.01), 9% (p=0.03) and 22% (p=0.004) increased mortality odds respectively. **Table 2** shows associations between significant immune cell subsets and mortality without adjustment for multiple comparisons from the above three models.

In the Model 3, after using Benjamini-Hochberg’s FDR procedure to account for multiple comparisons, total T cells (p=0.04), NK LO cells (p=0.04), and Neutrophils (p=0.04) remained associated with mortality. Non-significant results at 0.05 significance level from Model 3 are shown in **Supplementary Table 2**.

## 5 Discussion

In this study, we found that T cells and NK cells with low expression of CD56 were inversely associated with mortality while neutrophils were positively associated with mortality. In addition, we found myeloid dendritic cells to be nominally associated with a reduced odds of mortality, and CD4+ effector memory T cells and IgD- memory B cells to be nominally associated with increased mortality odds.

Several previous studies have shown a positive association between neutrophils and mortality and our study confirmed these previous findings (35, 36). The number of neutrophils are preserved in older adults though their phagocytic ability is impaired (37). Furthermore, since neutrophils are pro-inflammatory (38), higher numbers of neutrophils in older adults may increase the odds of mortality. NK cells cytotoxicity and IFN-γ production decreases in old age (39, 40), and low cytotoxicity is associated with increased morbidity and mortality (6). NK LO cells have significantly higher cytotoxicity than NK Hi cells (41). Hence, the observed inverse association with mortality was consistent with the biological activity of this NK cell subtype (17, 18). The absolute count of T cells decreases with age, and this decrease especially affects naïve subset (Tn). This alters the T cell repertoire, compromising their ability to mediate effective immune responses, and thus increasing the odds of mortality. The inverse association seen for total T cells in this study was in line with a previous study on hemodialysis patients (16). As immunosenescence is characterized by accumulation of memory and effector T cells (11, 42), a positive association between CD4+ effector memory T subset and mortality was consistent with the known distribution of this immune subset in older adults. Of note, we have previously shown that CMV seropositivity and not age was the predominant determinant of CD4+ effector memory T levels (43) suggesting that the association between some of the immune cell subsets and mortality may be primarily driven by environmental exposures as compared to age-related processes. Ramasubramanian et al. (13) showed that CD4+ naïve T cells had a strong inverse association with mortality, the same direction of association was seen in our analysis though the association was not significant (p=0.09) after adjustment for the covariates including comorbidity index and KDM-BA. Therefore, the association between CD4+ naïve T cell percentages and mortality may be explained, at least in part, by KDM-BA. Zhang et al. (15) showed that CD8+ T cells (Area Under the Curve (AUC)=0.786) were better predictors than NK cells (AUC=0.639) of mortality odds in patients with sepsis. However, we did not find any associations between CD8+ T cells and mortality in our study, though this discrepancy was likely due to study population differences as HRS participants were predominantly healthy individuals without sepsis.

A higher percentage of myeloid subset of dendritic cells was associated with reduced mortality, which was consistent with previous studies showing that dendritic cells mediate antitumor immune responses, and were used in immunotherapies and vaccinations that resulted in improved survival of cancer patients (44–46). This novel finding should be validated in other cohorts to demonstrate the generalizability of this association. IgD- Mem B cell remained positively associated with mortality in our study, and their level of significance became stronger upon additional adjustments. As B cell senescence is characterized by increase in apoptotic resistant memory B cells with reduced clonal expansion that leads to impaired antibody functioning and decreased opsonizing capabilities (7, 47, 48), the direction of association in this study was consistent with a previous study that showed that memory B cells were positively associated with cardiovascular mortality (49).

Higher chronological age, despite being a powerful predictor of mortality, is not a direct indicator of a person’s state of health. Previous studies using the National Health and Nutrition Examination Survey (NHANES) have shown that multi-dimensional aging metrics such as KDM-BA (5, 50) estimated mortality better than chronological age. Associations of biological age with all-cause mortality, type II diabetes, stroke, and cancer seen in Rotterdam study (51) indicates the prognostic value of biological age in determining disease occurrence. Though previous studies have demonstrated an association between immune cell subsets (13, 14, 16–18, 52) and mortality, we showed, for the first time, that the immune cells subsets remain independently associated with mortality even after adjustment for co-morbidities and KDM-BA, indicating that an aging immune system may affect mortality through mechanisms that are independent of age-related comorbidities and other multi-dimensional aging constructs that capture age-related multi-organ dysfunction. Systemic chronic inflammation contributes to a wide range of age-related health conditions such as ischemic heart disease, stroke, and cancer that remain the predominant causes of age-related morbidity and mortality (20). Though canonical markers of inflammation (IL-6, TNF-α, and CRP) have previously been shown to be associated with higher mortality in supercentenarians (53) and COVID-19 patients (54), there is an intricate interplay between immune cells and cytokine secretion (1, 6, 7, 20). When additionally adjusted for the inflammation latent variable, immune cell subsets remained strongly associated with mortality without substantial change in the observed hazard ratios. These findings suggest that other functions of immune cells independent of their ability to secrete various cytokines may be important mechanisms through which immunosenescence affects mortality odds. Thus, these findings indicate that the association between immune cell distribution and mortality is not influenced by biological aging, co-morbidities, and age-related inflammation. This independence, if validated in independent studies, suggests that the immune cells may be a novel biomarker for mortality prediction.

A major strength of this study was the adjustment for covariates including a multi-dimensional aging construct (KDM-BA), co-morbidities, and inflammatory biomarkers. Though several previous studies have shown associations of neutrophils (35, 36), lymphocyte subsets (13, 14, 16–18, 49, 52) and NK cells (17, 18) with mortality, this study extended those findings and provides evidence that features of an aging immune system are associated with mortality independent of concurrent age-related inflammation and diseases. In addition, this was the first study to evaluate and demonstrate an association between myeloid dendritic cells and mortality. A major limitation of this study is the short follow up time, restricting our ability to evaluate long term effects of immune cell distributions on mortality. Another limitation is our study evaluated only all-cause mortality and did not evaluate cause-specific mortality as those data are currently not available in HRS. In addition, delayed processing and cryopreservation of PBMCs may affect the percentages of cell subsets estimated. In a previous study, we have shown that the total numbers of dendritic cells remained unchanged under conditions used in this study (PBMCs cryopreserved after a delay of 48 hours) though the percentages of naïve CD8+ T cells, B cells and monocytes were slightly higher and the percentage of NK cells were slightly lower as compared to fresh blood samples (32).

## 6 Conclusion

This study demonstrated an association between individual immune cell subsets and mortality in a nationally representative sample of older adults (> 55 years) in the United States. Adaptive immune subsets (total T cells), innate subsets (NK cells (CD56LO) and neutrophils) were associated with 4-year mortality even after adjustment for biological age and chronic subclinical inflammation.

## Data availability statement

Publicly available datasets were analyzed in this study. This data can be found here: https://hrsdata.isr.umich.edu/data-products/public-survey-data and https://hrsdata.isr.umich.edu/data-products/sensitive-health.

## Ethics statement

The studies involving humans were approved by University of Minnesota Institutional Review Board. The studies were conducted in accordance with the local legislation and institutional requirements. Written informed consent for participation was not required from the participants or the participants’ legal guardians/next of kin in accordance with the national legislation and institutional requirements.

## Author contributions

GS: Conceptualization, Writing – review and editing, Formal Analysis, Investigation, Methodology, Software, Validation, Visualization, Writing – original draft. SV: Conceptualization, Validation, Writing – review and editing. AP: Validation, Writing – review and editing. EC: Writing – review and editing, Conceptualization, Funding acquisition. EK: Writing – review and editing, Validation. JF: Writing – review and editing, Conceptualization, Funding acquisition. WG: Writing – review and editing, Validation. HM: Validation, Writing – review and editing. BT: Writing – review and editing, Conceptualization, Funding acquisition, Project administration, Resources, Supervision.
