# Supplementary material for: Immune cells are associated with mortality: the Health and Retirement Study
Source: Front Immunol. 2023 Oct 20;14:1280144. doi: 10.3389/fimmu.2023.1280144 (PMC10623116; doi:10.3389/fimmu.2023.1280144)
Supplement: Supplementary file 1 [file DataSheet_1.pdf]

SUPPLEMENTARY MATERIALS

1 Supplementary Figures and Tables

1.1 Supplementary Tables

Supplementary Table 1: Markers used to determine 24 cell subsets using flow cytometry

CELL TYPE	MARKERS
T cells	CD3+ CD19
CD4+ T cells	CD3+ CD19- CD8- CD4+
CD4+ T cells: Central Memory	CD3+ CD19- CD8- CD4+ CD45RA- CCR7+ CD28+
CD4+ T cells: Effector	CD3+ CD19- CD8- CD4+ CD45RA+ CCR7- CD28
CD4+ T cells: Effector Memory	CD3+ CD19- CD8- CD4+ CD45RA- CCR7- CD28-
CD4+ T cells: Naïve	CD3+ CD19- CD8+ CD4+ CD45RA+ CCR7+ CD28+
CD8+ T cells	CD3+ CD19- CD8+ CD4
CD8+ T cells: Central Memory	CD3+ CD19- CD8+ CD4- CD45RA- CCR7+ CD28+
CD8+ T cells: Effector	CD3+ CD19- CD8+ CD4- CD45RA+ CCR7- CD28-
CD8+ T cells: Effector Memory	CD3+ CD19- CD8+ CD4- CD45RA- CCR7- CD28-
CD8+ T cells: Naïve	CD3+ CD19- CD8+ CD4- CD45RA+ CCR7+ CD28+
B cells	CD3- CD19+
IgD+ memory B cells	CD3- CD19+ IgD+ CD27+
IgD- memory B cells	CD3- CD19+ IgD- CD27+
Naive B cells	CD3- CD19+ IgD+ CD27-
Natural Killer (NK) Cells	CD3- CD19- CD20- CD14- CD16+ CD56+
NK Cells: CD56HI	CD3- CD19- CD20- CD14- CD16+ CD56HI
NK Cells: CD56LO	CD3- CD19- CD20- CD14- CD16+ CD56LO
Monocytes	CD3- CD19- CD20- CD14+
CD16- monocytes	CD3- CD19- CD20- CD14+ CD16-
CD16+ monocytes	CD3- CD19- CD20- CD14+ CD16+
Dendritic cells	CD3- CD19- CD20- CD14- HLA-DR+
Myeloid Dendritic cells (DC-M)	CD3- CD19- CD20- CD14- HLA-DR+ CD11c+ CD123-
Plasmacytoid Dendritic cells (DC-P)	CD3- CD19- CD20- CD14- HLA-DR+ CD11c- CD123+ PDC

Supplementary Table 2: Hazard ratios (95% confidence interval), p values from the fully adjusted model* for immune cells that are not significantly associated with mortality at 0.05 confidence level

Immune cell subsets	HR (95% CI)	p values
CD4+ T total	0.96 (0.86-1.07)	0.45
CD4+ Tcm	0.97 (0.87-1.08)	0.58
CD4+ Teff	1.08 (0.99-1.19)	0.09
CD8+ T total	0.99 (0.89-1.10)	0.84
CD8+ Tn	0.98 (0.84-1.14)	0.84
CD8+ Tcm	0.97 (0.87-1.07)	0.59
CD8+ Teff	1.03 (0.92-1.16)	0.57
CD8+ Tem	1.05 (0.95-1.16)	0.28
B total	1.06 (0.95-1.18)	0.29
Naive B	0.91 (0.83-1)	0.07
IgD+ mem B	1.09 (0.97-1.21)	0.13
NK total	0.94 (0.84-1.05)	0.28
NK Hi	1.07 (0.97-1.18)	0.17
Monocytes	1.05 (0.92-1.19)	0.48
CD16- monocytes	0.99 (0.90-1.11)	0.98
CD16+ monocytes	0.97 (0.90-1.05)	0.55
DC total	0.99 (0.87-1.14)	0.96
DC-P	1.05 (0.93-1.18)	0.38

*The above results were obtained after adjustment for chronological age, gender, race/ethnicity, smoking status, CMV seropositivity, BMI, KDM biological age, comorbidity index, and inflammation latent variable.

1.2 Supplementary Figures

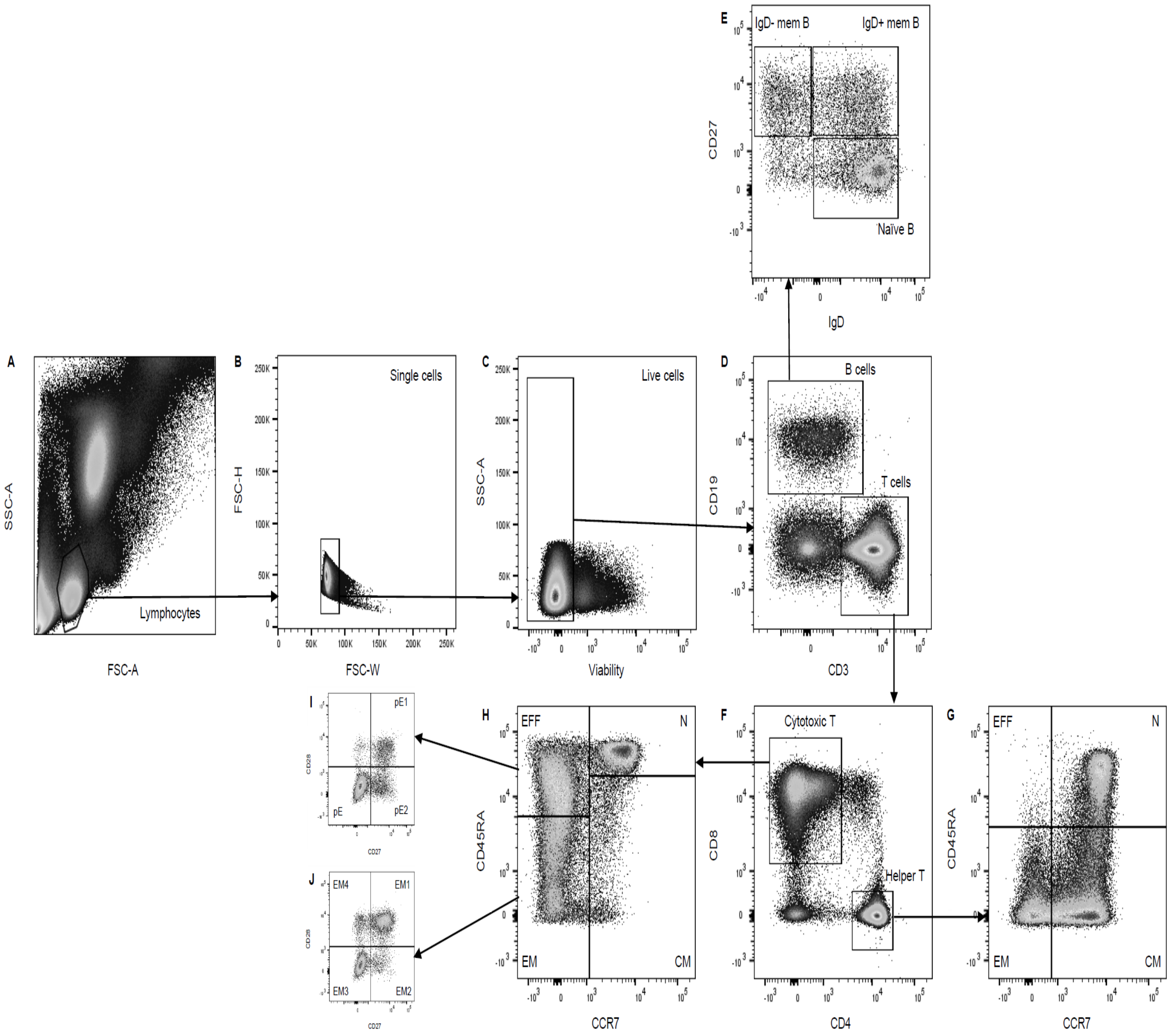

Supplementary Figure 1(a): Summary of gating strategy used for lymphocytes (Panel 1)

* The 24 immune cell subsets were measured using two flow cytometry panels.
Supplementary Figures 1(a) and 1(b) show the gating strategies used to identify the various immune cell subsets in Panels 1 and 2 respectively.

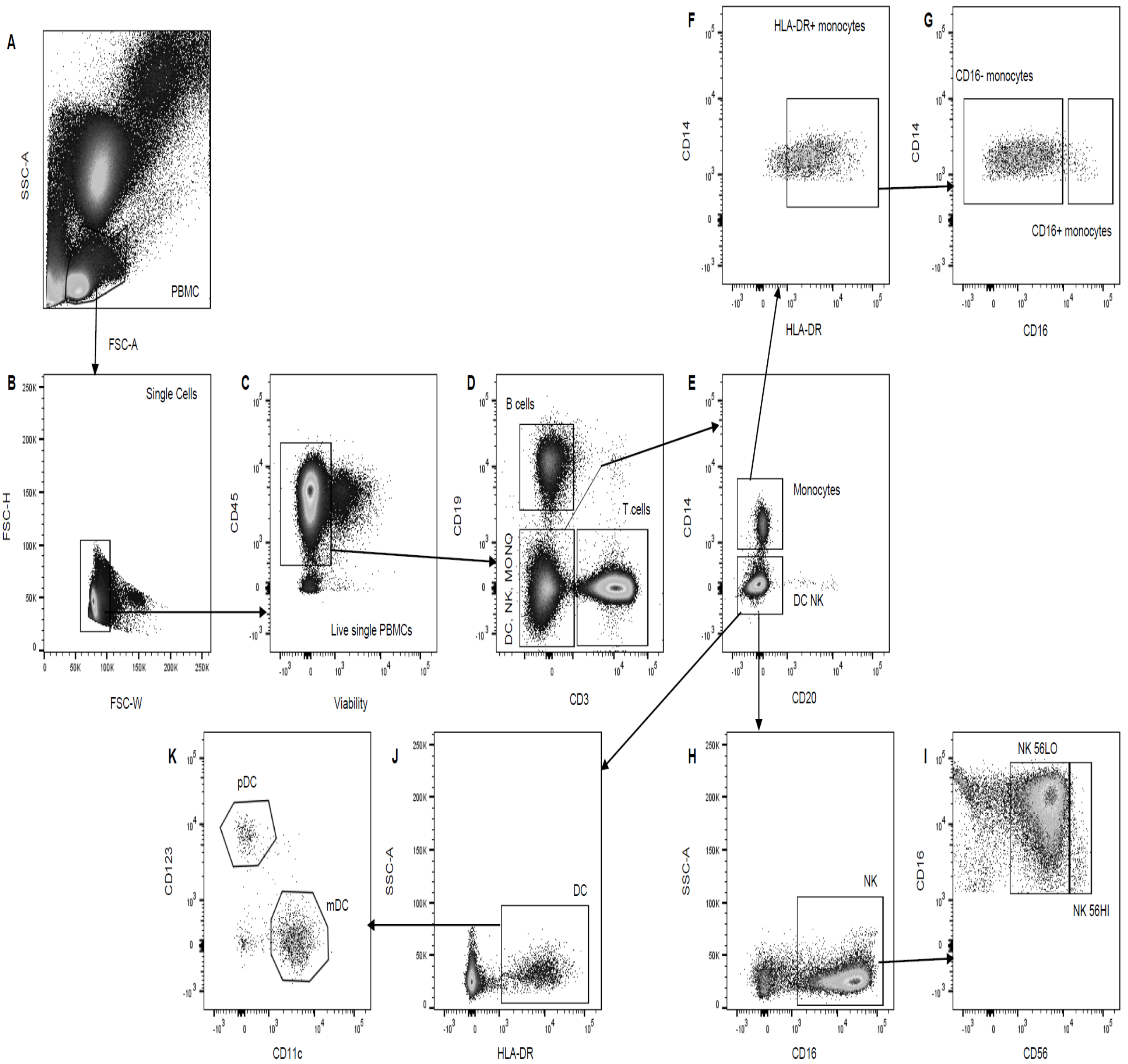

Supplementary Figure 1(b): Summary of gating strategy used for PBMC (Panel 2)

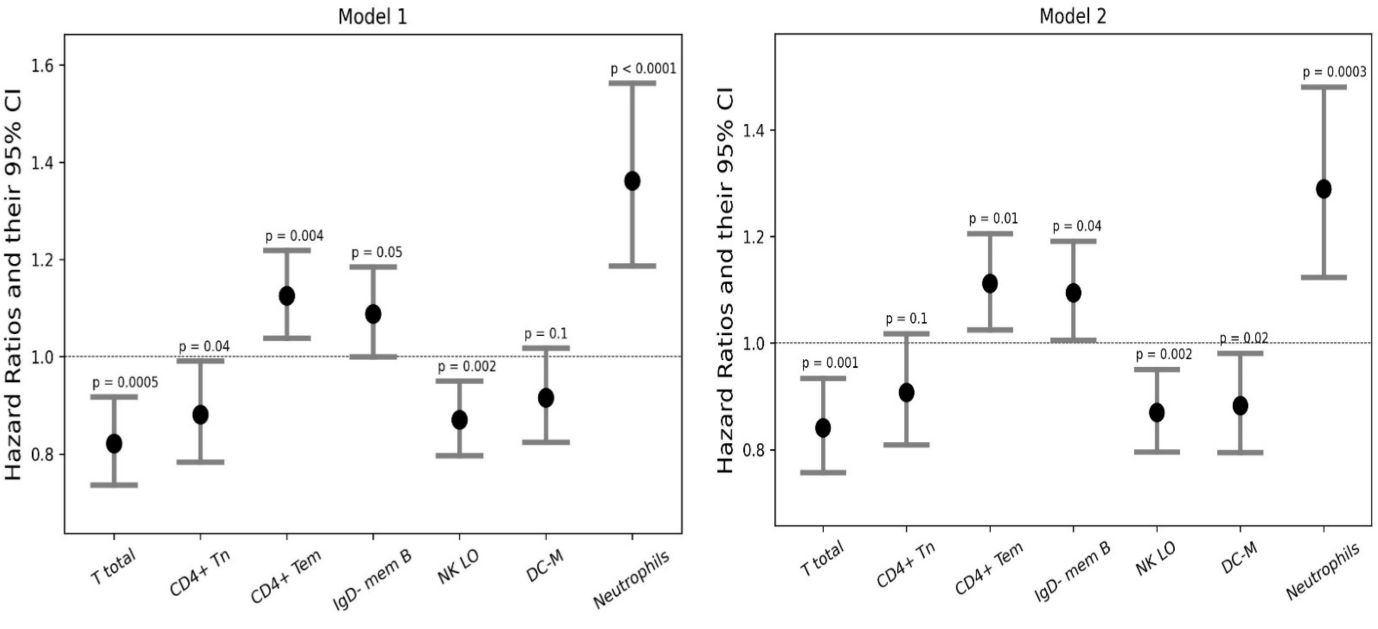

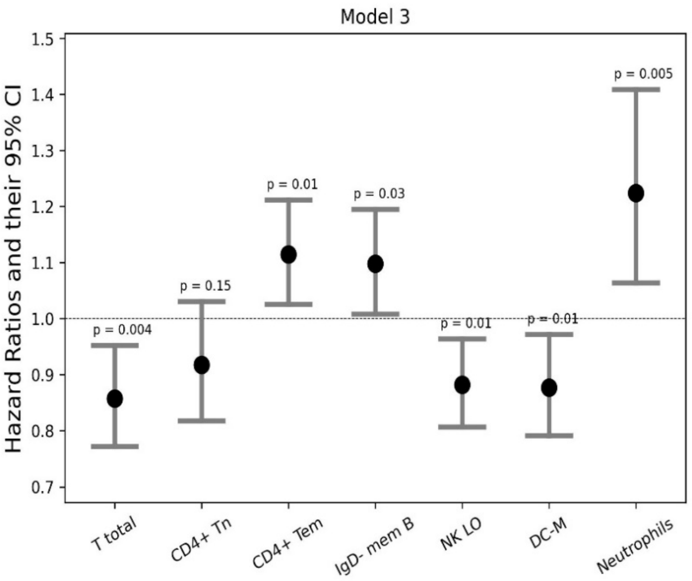

Model 1: Adjusted for chronological age, gender, race/ethnicity, smoking status, CMV seropositivity, and BMI

Model 2: Adjusted for covariates in Model 1 + KDM + comorbidity index

Model 3: Adjusted for covariates in Model 2 + inflammation latent variable

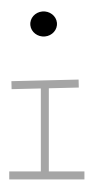

- Hazard ratio of the immune cell subset with respect to survival time (in days)
- 95% Confidence Interval of the hazard ratio

Supplementary Figure 2: Association between immune cell subsets and mortality in the Health and Retirement Study.

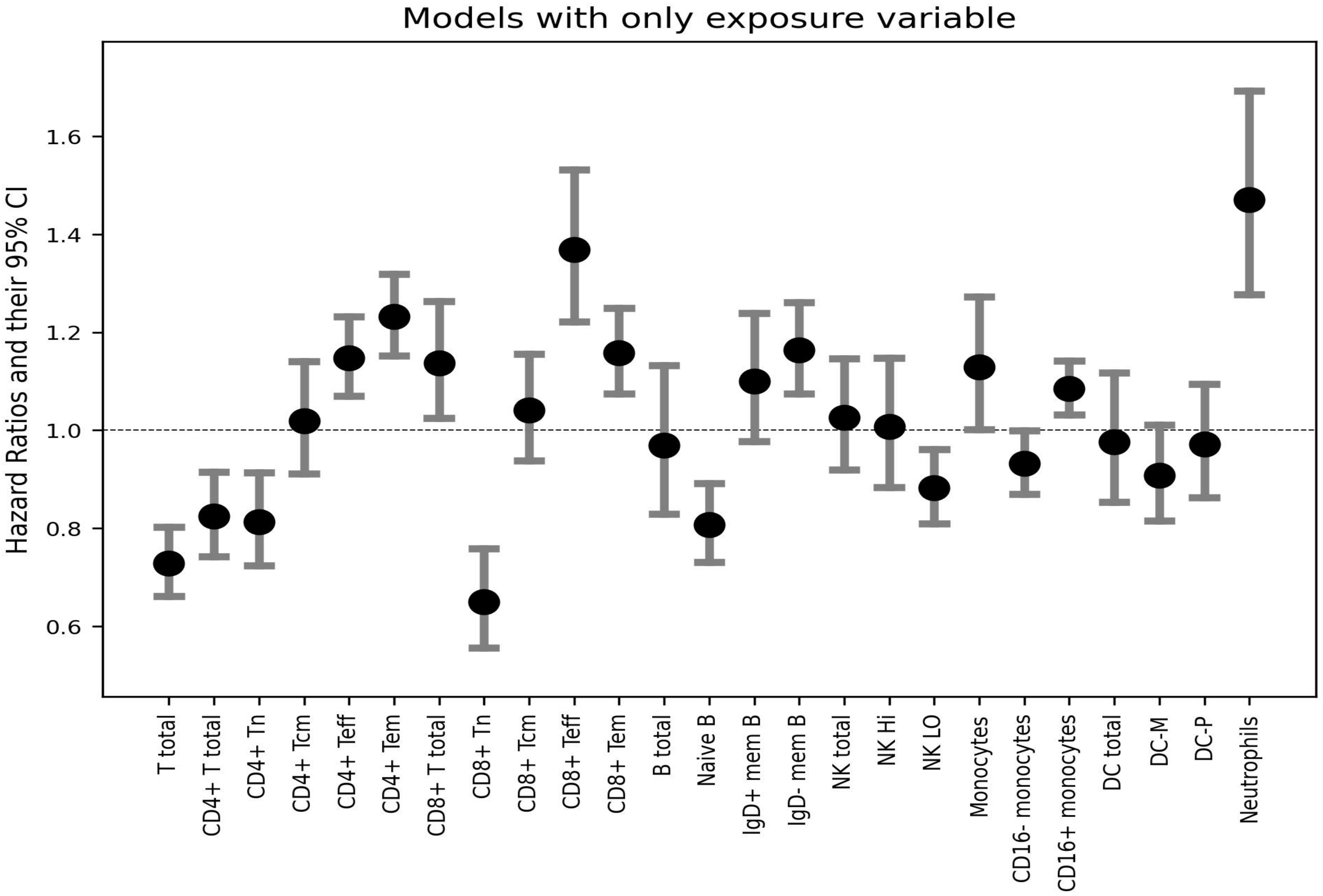

- Hazard ratio of the immune cell subset with respect to survival time (in days)

- 95% Confidence Interval of the hazard ratio

Supplementary Figure 3: Univariate analysis, hazard ratios and 95% confidence interval of immune cells against mortality without any adjustments

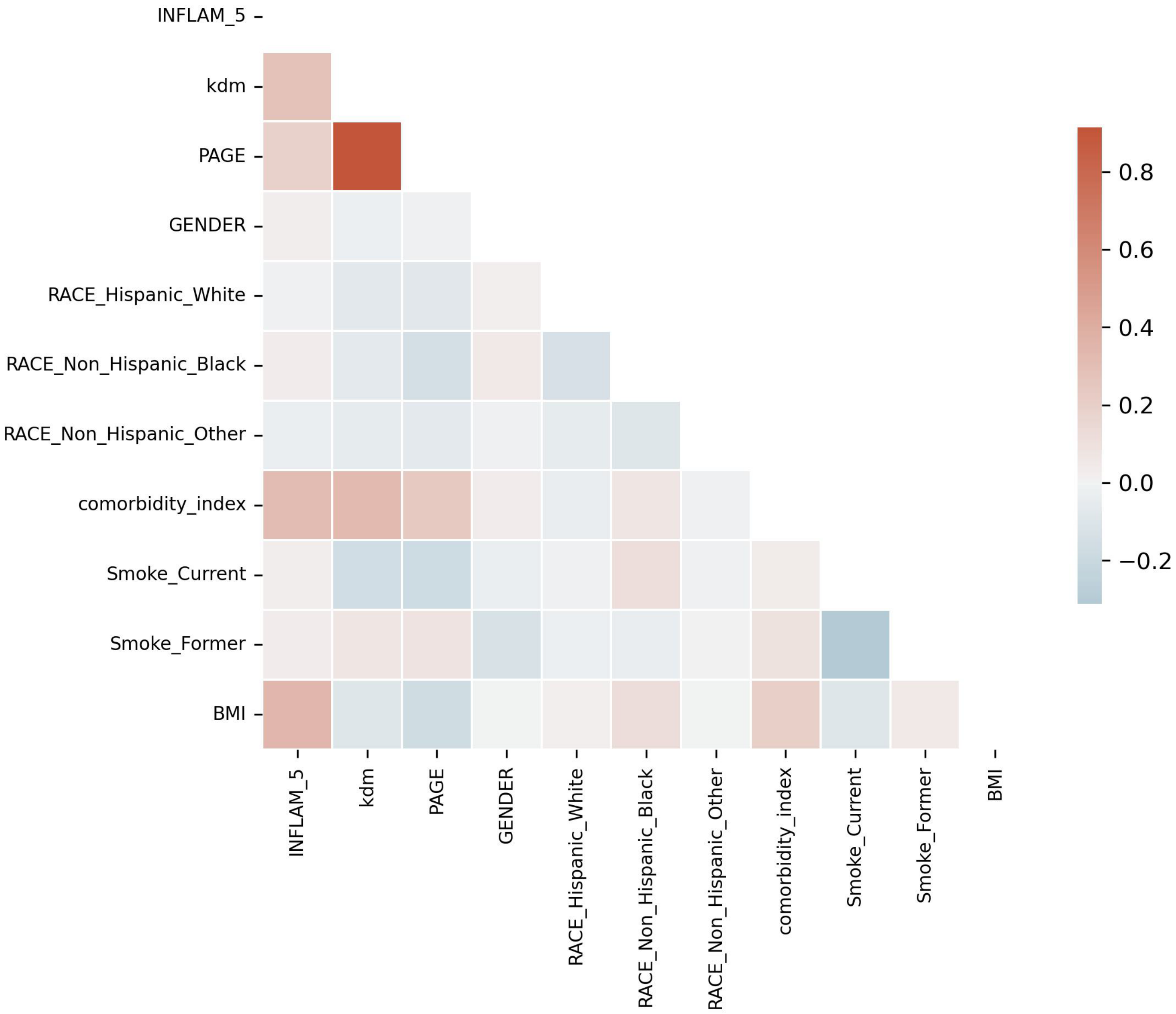

Supplementary Figure 4(a): Correlation matrix between the covariates.
KDM-BA is highly correlated with Chronological Age (PAGE), this is expected as Biological Age computation using Klemara-Doubal method (KDM) uses Chronological Age.

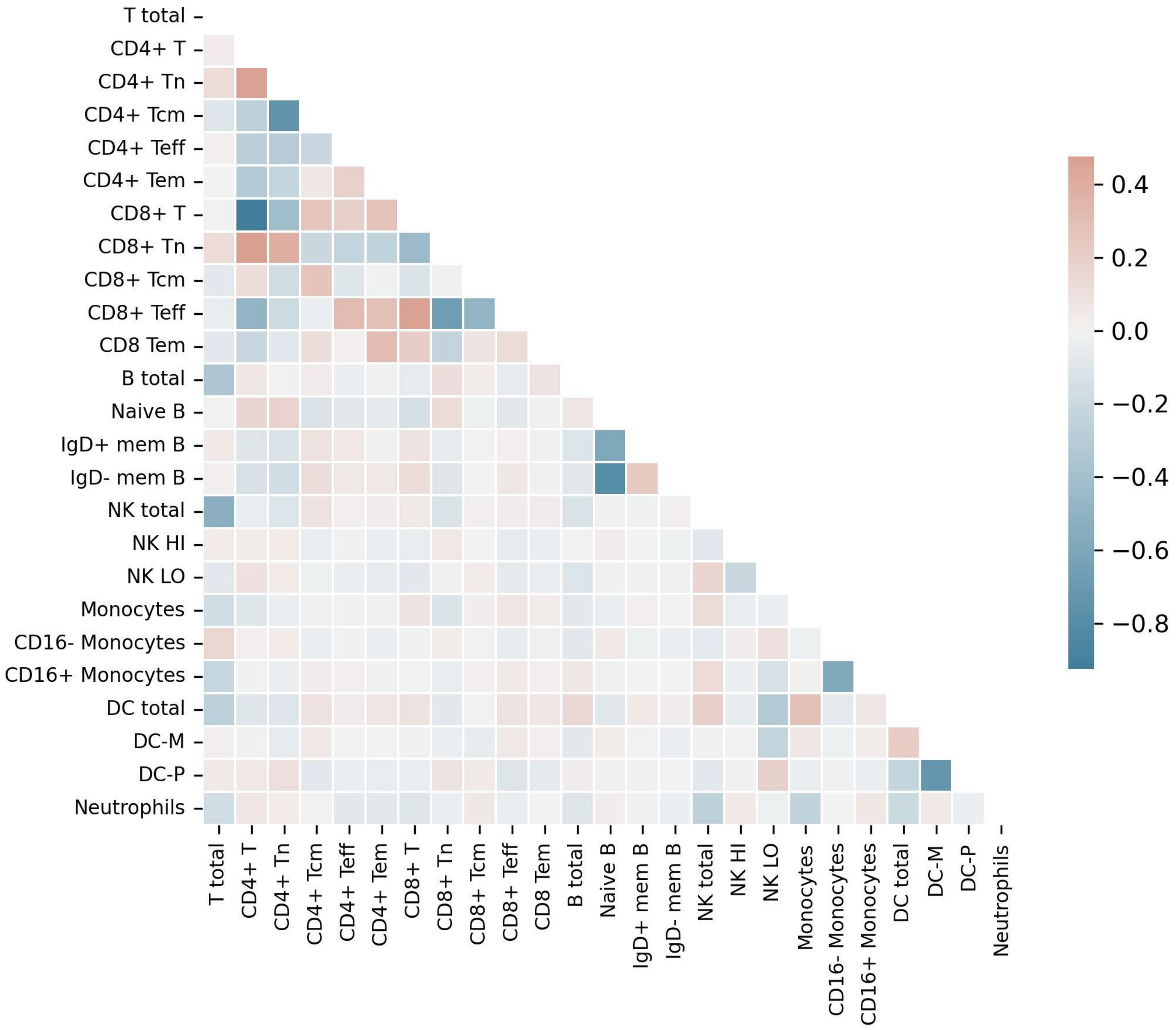

Supplementary Figure 4(b): Correlation matrix between the immune cell subsets.

The following subsets had levels of correlation > 0.6: myeloid dendritic cells were negatively correlated (r=-0.73) with plasmacytoid dendritic cells; CD4+ T cells were negatively correlated (r=-0.92) with CD8+ T cells; CD8+ naive T cells were negatively correlated (r=-0.67) with CD8+ effector cells; CD4+ naive T cells were negatively correlated (r=-0.74) with CD4+ central memory T cells; and naïve B cells were negatively correlated (r=-0.80) with IgD- memory B cells.

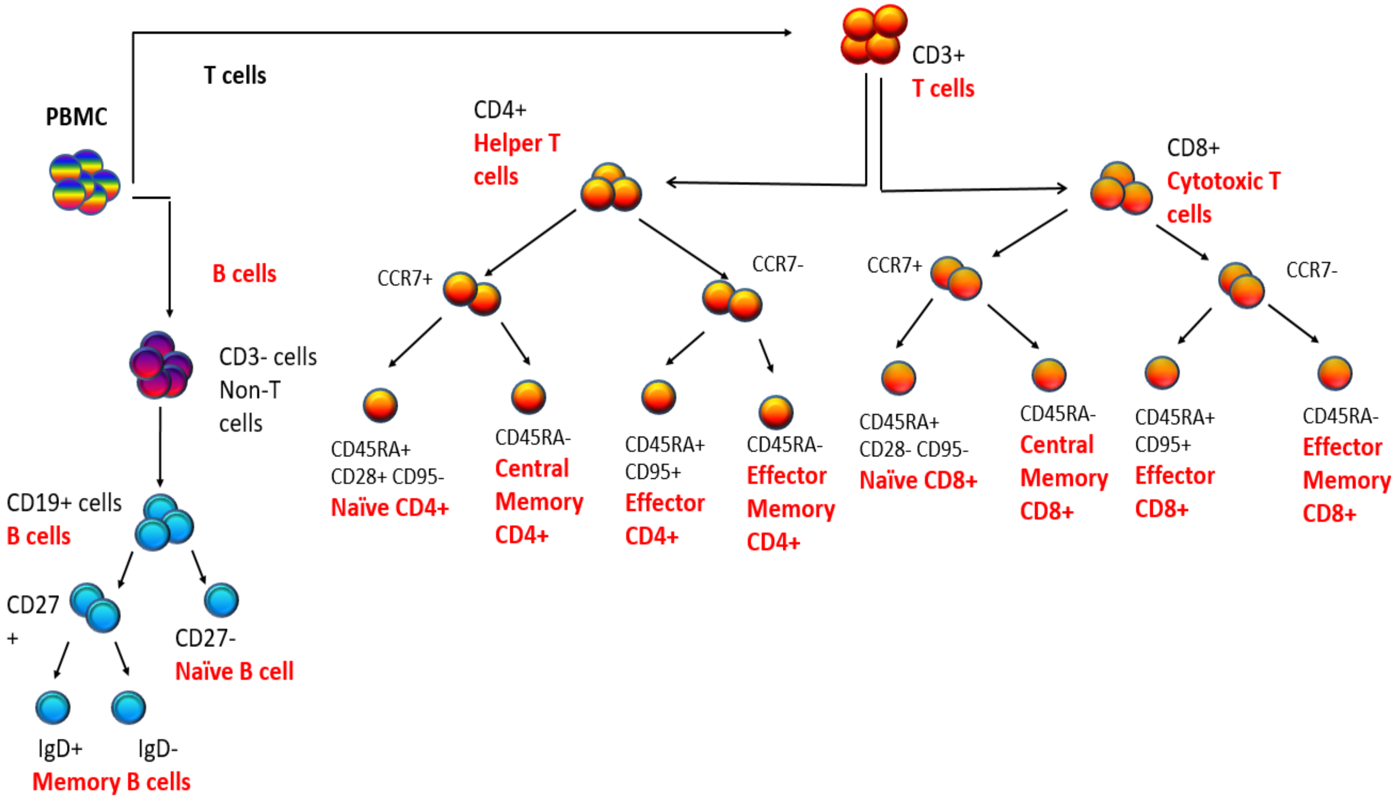

Supplementary Figure 5(a): Schematic representation of adaptive immune cell subsets (T cells and B cells) identified by immunophenotyping.

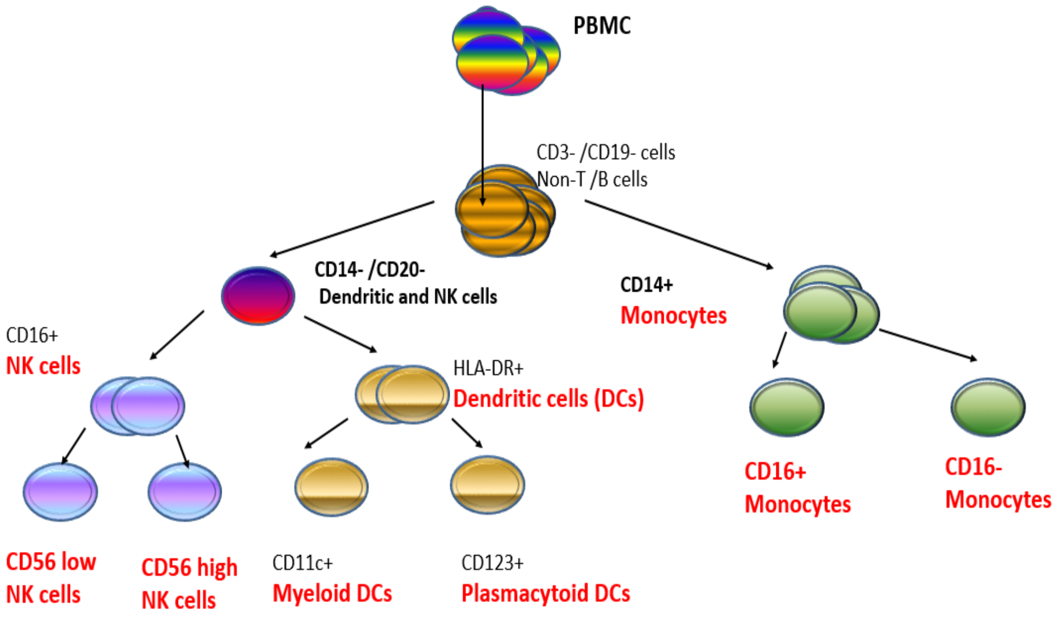

Supplementary Figure 5(b): Schematic representation of innate immune cell subsets (monocytes, NK cells, and dendritic cells) identified by immunophenotyping.
